# Wide Excision of a Retroperitoneal Liposarcoma with En Bloc Ureterectomy and Renal Salvage by Autotransplantation

**DOI:** 10.1155/2019/9725169

**Published:** 2019-12-11

**Authors:** Siegfredo R. Paloyo, Arjel D. Ramirez, Ferri P. David-Paloyo, Rodney B. Dofitas

**Affiliations:** ^1^Department of Surgery, University of the Philippines-Philippine General Hospital, Manila, Philippines; ^2^College of Medicine, University of the Philippines-Manila, Manila, Philippines

## Abstract

Liposarcoma is a malignant mesenchymal neoplasm composed of adipose tissue with varying degrees of atypia. These tumors grow slowly and may reach an enormous size particularly if located in the retroperitoneum. We report a 40-year-old male with a 6-month history of gradual abdominal enlargement. Computed tomography (CT) of the abdomen showed a huge retroperitoneal mass with characteristic features consistent with liposarcoma. On laparotomy, the mass was noted to be encasing the right ureter for which a wide excision with en bloc ureterectomy and subsequent renal autotransplantation for organ preservation was done. Post-operative course was uneventful with excellent outcome after 6 months of follow-up. Final histopathologic diagnosis was low-grade, well differentiated liposarcoma, which has favorable prognosis following radical surgery. This was the first report of such a case in the Philippines.

## 1. Introduction

Retroperitoneal liposarcoma is a rare tumor and exhibits considerable histological heterogeneity. The Importance of adequate staging and grading in guiding the management approach of such cases cannot be overemphasized. Surgery is the mainstay of treatment and a macroscopically complete resection gives the patient the best chance of prolonged recurrence free survival and over-all survival. We herein report our first case of retroperitoneal liposarcoma for which radical surgery was performed followed by renal autotransplantation to salvage the organ. This case highlights the importance and application of oncologic surgical principles while giving equal importance to surgical technique.

## 2. Case Report

A 40-year-old male, with no known comorbidities, came in due to a 6-month history of a gradually enlarging abdomen with associated weight loss and early satiety. He complained of occasional dyspnea especially in a recumbent position and was able to do activities of daily living with some limitations. There were no other associated symptoms noted. On physical examination, there was a huge, movable, firm mass occupying the distended, nontender abdomen.

**Figure 1:**
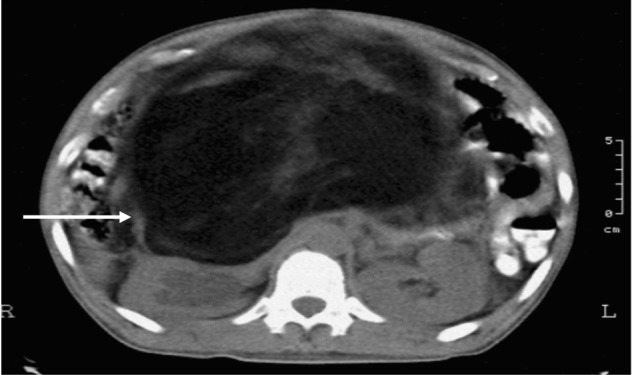
Retroperitoneal mass (white arrow) with fat and soft tissue component occupying almost the entire abdominal cavity pressing unto the right kidney.

Contrast-enhanced CT scan showed a soft tissue mass at the midline measuring 47 × 34 × 17 cm originating from the right retroperitoneum, predominantly fat with interspersed areas of solid soft tissue component, with note of lateral displacement of the bowel segments and right pelvic ureter ectasia. It shows a clear plane from the aorta while the inferior vena cava is compressed (Figure 1).

**Figure 2:**
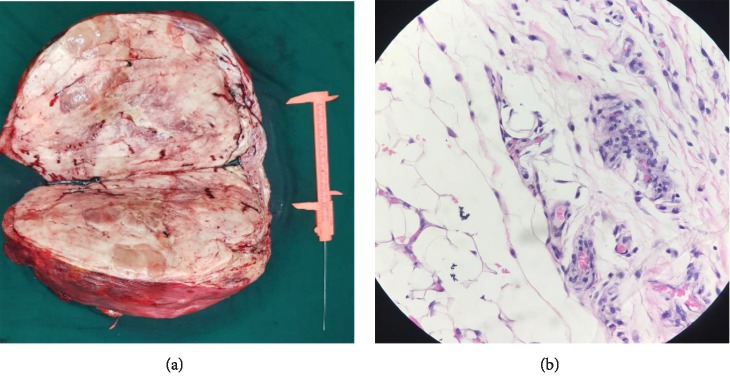
Macroscopic and microscopic view of the tumor. (a) Gross view of the tumor: 47 × 34 × 17 cm and weighs 11 kg. (b) Histopathology showing a well-differentiated liposarcoma with scattered bizarre stromal cells and marked nuclear hyperchromasia (hematoxylin & eosin stain, 400x).

**Figure 3:**
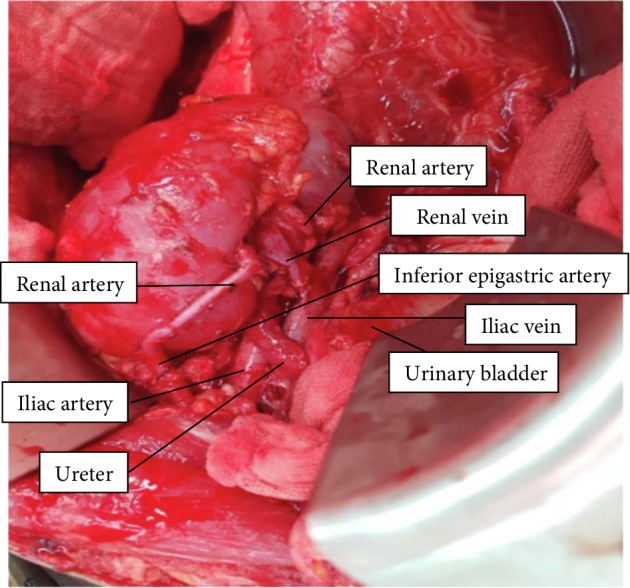
The autotransplanted right kidney.

Intraoperatively, the mass was encasing the right ureter for more than two-thirds of its length while the aorta, inferior vena cava, iliac vessels, and the right kidney were free from attachments to the tumor. Wide excision with en bloc right ureterectomy was performed, leaving approximately 3 cm of the proximal 3^rd^ of the ureter (Figure 2). We then proceeded with renal autotransplantation noting two veins and three arteries on the right kidney. After adequate flushing at the back table, the kidney was placed at the right iliac fossa anastomosing one of the renal veins (the other vein was ligated) and two renal arteries to the external iliac vein and artery, respectively. The third renal artery was then anastomosed to the inferior epigastric artery while the remnant ureter was re-implanted to the anterolateral bladder wall (Figure 3). Warm ischemia time was 26 minutes.

Post operative course was uneventful. Renal Doppler ultrasound showed good flow on all vessels with a nuclear scan showing adequate cortical tracer uptake on the autotransplanted kidney. Final histopathologic diagnosis was low grade, well-differentiated liposarcoma with negative margins of resection.

## 3. Discussion

Soft tissue sarcomas are a heterogeneous group of tumors with varying prognosis representing less than 1% of all cancers with a global incidence of 5 per 100,000 population per year [1]. The retroperitoneum provides an environment in which sarcomas can easily attain extensive growth before they become symptomatic. Around 45−50% of retroperitoneal sarcomas are liposarcomas with well-differentiated (also known as atypical lipomatous tumor) histology, accounting for most cases. Other histological subtypes include myxoid (round cell), mixed-type, pleomorphic, and dedifferentiated. Well-differentiated liposarcomas are indolent, well-circumscribed lesions characterized by rapidly growing mature-appearing adipocytes with atypical hyperchromatic nuclei scattered between adipocytes or within fibrous septa and multivacuolated lipoblasts.

Surgical resection with negative margins, offers the best prognosis with a 5-year overall survival of 80% for well-differentiated types although half of these patients will demonstrate locally recurrent disease within 5 years. Evans et al. reported a 100% recurrence rate in 19 patients who were followed for at least 10 years, which may suggest higher rates with longer follow-up [2]. Histology, tumor grade and the ability to completely resect the tumor remain important predictors of recurrence. The extent of disease may at times require enbloc resection of contiguous structures such as the kidneys, the ureters, and the intestines. Increased chance of recurrence albeit low, the risk of metastasis argues for adjuvant therapy. However, the role and effectiveness of such an approach, to increase local control and improve quality of life, has been highly debated. Currently, there are no consensus guidelines with regards to the use of radiation therapy for well-differentiated liposarcomas, except for recurrent disease.

Russo et al. demonstrated that there is no direct renal invasion in the majority (73%) of their cases, consequently, renal sparing resections can be performed [3]. However, in cases where there is involvement of adjacent structures, renal autotransplantation may be an option particularly if the expertise is available. Hardy first demonstrated its use for high ureteral injuries in 1963 [2]. Since then, autotransplantation has been found to be a safe and effective surgical procedure for the treatment of complex urologic conditions. Rare as it is, it is even rarer to be an option as a salvage procedure after tumor extirpation, the rationale being organ preservation. It served as an alternative treatment modality because of the substantial loss of ureter length due to tumor extension in this case. There is a paucity of experience in the literature; in fact, we only found 2 other similar case reports. Shchepotin et al. performed autotransplantation for liposarcomas involving likewise the ureter while the other was the renal vein [4]. Both patients had good renal function and negative tumor recurrence at one year follow up. Another report by Bansal et al. describes a patient where the ureter went through the capsule of the tumor with involvement of a segment of ileum, necessitating enbloc resection and anastomosis with autotransplantation [5]. To our knowledge, this is the first documented case in the Philippines.

Additional technical challenges added interest in this case, as we found 2 veins and 3 arteries. Ligation of one of the renal veins was inconsequential as tributaries communicate within the kidney. Furthermore, the use of the inferior epigastric artery as an ancillary inflow source has been well established in grafts with multiple arteries yielding excellent patency rates. Although it increased our ischemia time, it was not detrimental to the kidney function as evidenced by the nuclear scan.

In conclusion, retroperitoneal liposarcomas are locally aggressive tumors. Complete surgical excision with negative margins provides the best chance of attaining disease-free survival, however, in the process of obtaining oncologic margins, equal effort in organ preservation should be employed at times. Such cases may benefit timely referral to high volume centers where multidisciplinary technical expertise is available.
